# Profiling lower extremity injuries sustained in a state police population: a retrospective cohort study

**DOI:** 10.1186/s12891-021-03986-3

**Published:** 2021-01-26

**Authors:** Kate Lyons, Mick Stierli, Ben Hinton, Rodney Pope, Robin Orr

**Affiliations:** 1 Bond Institute of Health and Sport, Bond University, Gold Coast, QLD 4229 Australia; 2 NSW Police Force Reconditioning Program, Surry Hills, NSW 2010 Australia; 3 Tactical Research Unit, Bond University, Gold Coast, QLD 4229 Australia; 4 School of Community Health, Charles Sturt University, Albury, NSW 2640 Australia

**Keywords:** Law enforcement, Police, Injuries, Lower extremity, Musculoskeletal

## Abstract

**Background:**

Tactical populations, such as military, firefighter and law enforcement populations, are known to suffer a relatively high number of musculoskeletal injuries, with the lower extremity of notable concern. The aim of this retrospective cohort study was to determine the profile of lower extremity musculoskeletal injuries within a state police agency.

**Methods:**

Injury data were collected by an Australian state police force over a 7-year period (2009–2016) and records not meeting the definition for lower extremity musculoskeletal injury were excluded. Statistical analyses were descriptive, with frequencies, means and standard deviations calculated where applicable. Chi-square analysis was performed to compare injury profiles by gender. Ethics approval was granted by Bond University Human Research Ethics Committee (Research Protocol 15360).

**Results:**

Of the initial 65,579 incident records, 12,452 (19%) related to lower extremity musculoskeletal injuries. The knee was the most commonly injured site (31.4%) with sprains/strains (42.3%) the most common nature of injury and arresting offenders (24.2%) the most common activity at time of injury. Slips/trips/falls (37.8%) was found to be the most common cause of injury. Variations were found between genders, most notably within the injury activity (*p* < .001). 27.1% of male officers were injured when arresting offenders compared to 16.5% for female officers. Walking/running contributed to 17.9% of female officer incidents compared to 9.3% for male officers. The mean number of hours worked prior to injury occurrence was 6.00 ± 3.56 h with significantly more injuries occurring in the middle third of the shift (4.34–8.67 h, *p* < 0.001).

**Conclusions:**

While the proportion of injuries that affected the lower extremity was lower for police, the leading sites of injuries (knees and ankles) were similar to those of military and fire and rescue populations. Variations between genders suggest there may need to be differences in return-to-work rehabilitation.

## Background

The Centre for Disease Control and Prevention defines musculoskeletal (MSK) injuries as soft-tissue injuries affecting the muscles, nerves, tendons, joints and cartilage, caused by repetitive motions either sudden or sustained, external forces, awkward positions, or vibrations [1]. In the literature, MSK injuries have also been defined as any injury to a joint, ligament, tendon, bone or muscle [2]. MSK disorders or injuries are common in occupational settings [3–5]. Tactical populations, such as military (inclusive of Army, Navy and Airforce), firefighter (inclusive of fire and rescue) and law enforcement (inclusive of police and custodial) populations, are known to suffer a relatively high number of MSK injuries [6–9]. For example, MSK injuries, to the lower extremity alone, have been reported to make up between 32% [6] and 43% [9] of injuries within military populations in Australia and New Zealand respectively. In Australian fire and rescue personnel, MSK injuries were found to contribute 66.5% of all injuries [7]. Among these injuries, the knee and ankle were the most commonly injured body sites [7]. In Australian law enforcement populations detailed MSK injury data is limited. However, Australia law enforcement injuries, in general, have been reported to occur at a rate of 3023 injuries per 100,000 officers [10]. More recently, an audit of work hours lost due to injury reported an average of 78 lost hours per officer during 2018–19, up 14 lost hours per officer from the preceding year [11]. Noting that most literature reports incidence rates of injuries without identifying the proportions of those injuries that are MSK injuries [1, 12–18], it is not surprising that it has been reported that there is insufficient research and data relating to occupational injuries affecting law enforcement officers (LEO) [19].

One known cause of MSK injuries associated with tactical professions is load carriage [6, 9, 20–23]. Like military personnel and fire fighters, LEO are required to wear and carry loads [24, 25], which can range from 3 kg to 15 kg [26] in general duties officers, and from 22 kg [27] to 40 kg [28] in specialist officers (e.g. riot squad officers, special weapons and tactics teams, etc.). Typically these loads, which consists of body armour, duty belts, baton, sidearm, handcuffs, etc. [25], are worn by LEO when they are conducting manual handling tasks, undertaking offensive or self-defence manoeuvres while arresting suspects or non-compliant offenders, or undertaking foot pursuits of suspects [28, 29]. In addition, LEO can be required to perform these tasks, whilst wearing these loads, in variable situations that are arduous, unpredictable and can be life threatening in some circumstances [30]. Physical stress, including that from load carriage, heavy lifting, awkward positioning and environmental stressors, has been shown to increase the risk of sustaining an occupational injury [31]. On this basis, it is not surprising that LEO have a higher MSK injury risk when compared to the general population [12].

MSK injuries in the law enforcement work force can have considerable downstream effects, beyond lost productivity. For example, while studies have demonstrated that productivity, in terms of quality/quantity of work and presenteeism, can be adversely impacted by MSK injury [32, 33], loss of personnel due to MSK injuries, particularly those leading to long term or permanent disability, can require backfilling of positions, the recruitment and training of new personnel, and an increased occupational workload for remaining staff members [33]. For LEOs this could also mean increases in overtime to cover for those who are injured and therefore greater exposure of personnel to occupational risk factors.

Despite the high risks of injury to LEO and the potential downstream impacts of these injuries, research investigating injuries within law enforcement populations, although of fair quality, is limited [34]. A recent review by Lyons, et al. [34] found that the majority of studies in law enforcement contexts that have investigated injuries included only male participants. Understanding differences in injury presentations between male and female officers is of importance as research has shown that, for examples, male and female tactical personnel can suffer from different lower limb musculoskeletal injuries [35] and that lower limb interventions to address gender-specific injuries are effective [36]. Furthermore, the review found that there were no standard injury definitions, with many of the included studies failing to include any definition at all [34]. In addition, many of the included studies in the review only reported body site of injury in terms of broad locations, for example ‘lower extremity’ rather than a specific site such as the knee [34]. To address these gaps in the literature and to further inform future injury mitigation strategies, the aim of this retrospective cohort study was to profile lower extremity MSK injuries within a state police agency.

## Methods

Retrospective injury data, collected by an Australian state police department as part of their standard operating procedures for recording workplace incidents and injuries, were provided to the research team for analysis. The data consisted of 7 years of non-identifiable workplace injury records compiled from mandated incident reporting forms which recorded all workplace injuries submitted between 1st July 2009 and 30th June 2016 with data input to the system by qualified and task dedicated personnel. Population data were also provided by this Australian state police department for the financial years 2009/2010–2015/2016, to enable calculation of injury rates.

For this study, workplace injury was defined as ‘harm to the body which occurred as a result of energy applied to the body whilst on duty’. This definition was derived from a previous literature review [34] of the injuries sustained by LEO and fitted to the reporting context of this population. MSK injuries were defined by the CDC definition provided above [1]. Lower extremity was defined to include toes, foot, ankle, knee, groin, hip, upper leg and lower leg. The pelvis was not included, in order to allow for comparison with findings from other tactical population research, which excluded the pelvis [37]. The data were refined in a manner that reflected these definitions, by employing a tiered exclusion process. First, injured body sites which did not meet the lower extremity criteria were removed, and this was followed by removal of records of non-MSK injuries. The data were then manually checked to ensure there were no ineligible entries remaining.

Ethics approval for the research was granted by the Bond University Human Research Ethics Committee (BUHREC) (Research Protocol 15360), with gatekeeper approvals also obtained from the police force from which the data were drawn.

Upon receiving the injury data, most of the data had already been systematically coded by the state police agency as part of their data entry procedure. For the data variables which were not coded (e.g. incident type), a predetermined numerical code was assigned in a systematic and standardised fashion (e.g. Male = 1, Female = 2 etc.), to allow for interpretation by the analysis software. The coding systems for these originally uncoded variables were generated by the researchers, with a code sheet created to ensure consistency in records categorisation. The coded data were then imported into IBM SPSS (version 24) [38] for analysis.

Statistical analysis of the data were initially completed descriptively, which enabled frequencies to be determined and means with standard deviations (SD) to be calculated for appropriate variables, such as lengths of shifts, hours worked 7 days prior to injury, and hours worked prior to incident occurrence. Chi-square tests of independence were then performed to compare frequencies from key descriptive variables between genders, with Cramer’s V then calculated to determine the strength of any significant association with the alpha level set at < .001, a priori. Shift lengths were divided into three categories - beginning, middle and closing third of shift - with a chi-square goodness of fit conducted to determine differences in proportions of injuries reported in each third of the shift. The alpha level was deliberately stringent to account for the large number of chi-square analyses conducted and to control family-wise error rates.

Incidence rates were calculated per annum using the following formula, where the total number of injuries and the annual population numbers used were either all, female only or male only, to determine the incidence rates for each. The total number of injuries and the sum of annual populations were each divided by seven because the data covered a seven-year period in each case and needed to be converted to average annual numbers.

$$ \mathrm{Annual}\ \mathrm{MSK}\ \mathrm{injury}\ \mathrm{incidence}\kern0.5em =\frac{\left(\mathrm{total}\kern0.5em \mathrm{number}\kern0.5em \mathrm{of}\kern0.5em \mathrm{injuries}\kern0.5em \div \kern0.5em 7\right)}{\left(\mathrm{sum}\kern0.5em \mathrm{of}\kern0.5em \mathrm{annual}\kern0.5em \mathrm{population}\kern0.5em \div \kern0.5em 7\right)}\times \kern0.5em 100\kern0.5em \mathrm{injuries}\kern0.5em \mathrm{per}\kern0.5em 100\kern0.5em \mathrm{per}\mathrm{sonnel} $$

## Results

Population sizes for LEO serving in the Australian state police department that was the focus of this study over the years included in this study are detailed in Table 1, which includes a gender breakdown of the annual populations.

**Table 1:** Police department population sizes (sworn members), by financial year, from 1st July 2009 to 30th June 2016

^a^Financial Year (FY)	Total LEO number	Male officer number (%)	Female officer number (%)
FY09–10	16,112	11,814 (73.3%)	4298 (26.7%)
FY10–11	16,033	11,746 (73.3%)	4287 (26.7%)
FY11–12	16,293	11,918 (73.1%)	4375 (26.9%)
FY12–13	16,384	11,962 (73.0%)	4422 (27.0%)
FY13–14	16,692	12,151 (72.8%)	4541 (27.2%)
FY14–15	16,659	12,106 (72.7%)	4553 (27.3%)
FY15–16	16,677	12,132 (72.7%)	4545 (27.3%)

^a^FY is defined as the period between 1 July of 1 year and 30 June of the next year

In total, 65,579 workplace injuries were reported. On implementation of the first data exclusion criterion, records (*n* = 48,959: 75%) which did not identify the lower extremity as the body site of injury were removed from the total data set, leaving 16,620 (25%) records pertaining to lower extremity injuries. On implementation of the second data exclusion criterion, records (*n* = 4168: 6%) of non MSK injuries were removed from this data set, leaving 12,452 (19%) records pertaining to lower extremity MSK injuries; 9055 (72.7%) involved male officers and 3397 (27.3%) involved female officers. Therefore, the overall MSK lower extremity incidence rates were 10.8 injuries per 100 personnel/year overall, 10.8 injuries per 100 personnel/year for male LEO, and 11.0 injuries per 100 personnel/year for female LEO.

Due to potential entry errors 99 (0.8%) entries were removed, as shift lengths were reported as being longer than 13 h and thus considered by data providers to be a potential error, and a further 89 reports did not record shift length. Based on the remaining 12,264 records, the mean number of hours worked per shift prior to each reported injury was 6.00 (±3.56) hours with 36.5% occurring < 4.34 h, 37.5% between 4.34–8.67 h and 26.0% occurring between 8.68 and 13 h. A significantly greater number of injuries occurred during the middle third of the shift (χ^2^(2) = 298.221, *p* < .001).

**Fig. 1:**
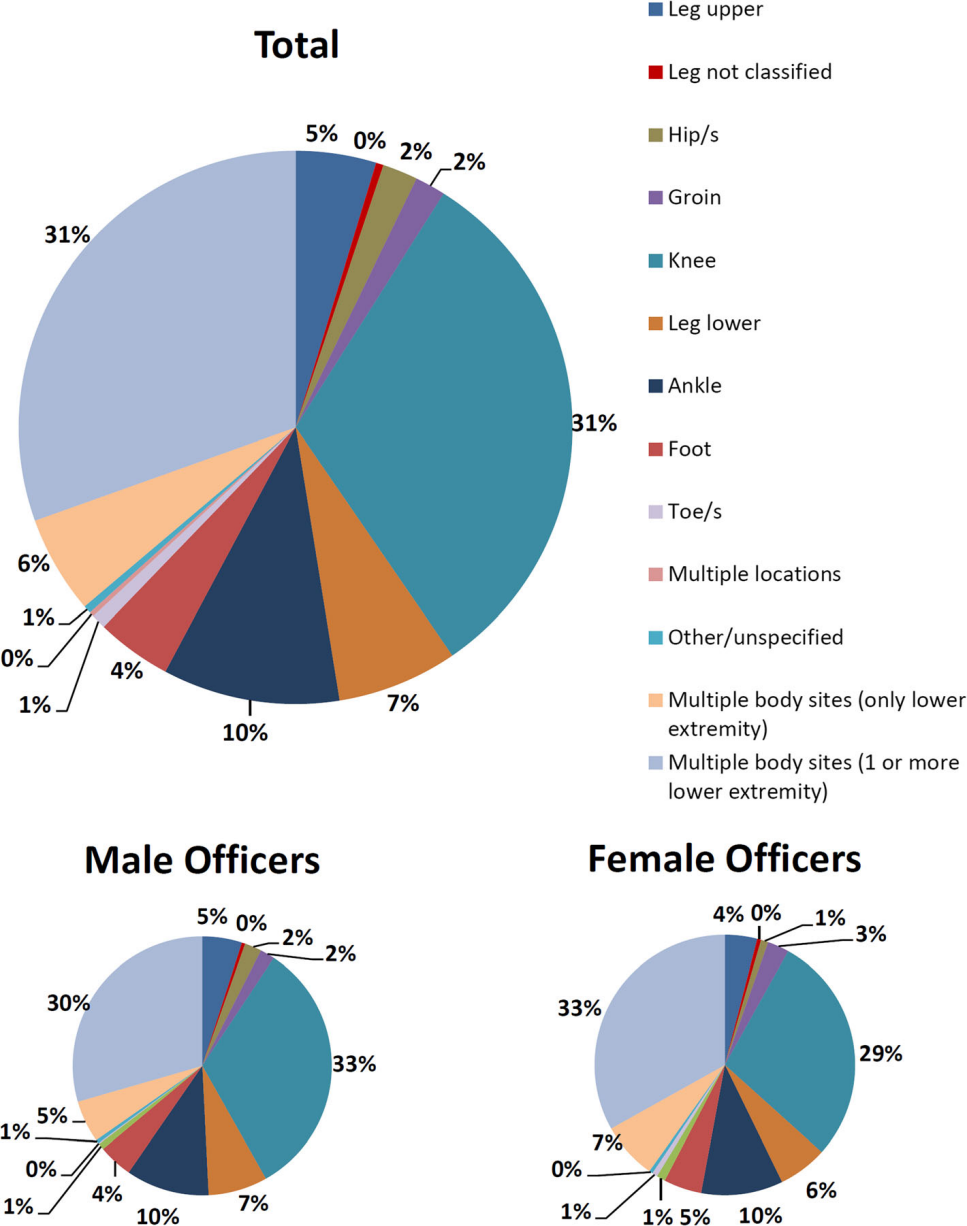
Pie charts showing the distributions of injured body sites across all injured personnel (%) and by gender

The top three injured body site categories were found to be the ‘knee’ (*n* = 3915, 31%), ‘multiple body sites including lower extremity’ (*n* = 3794, 30%), and the ‘ankle’ (*n* = 1287, 10%). The full injury body site distribution is shown in Fig. 1, with male and female officer comparisons also provided.

The most common nature of injury in the LEO was ‘sprains/strains’ (*n* = 5259, 42%), followed by ‘multiple natures including MSK’ (*n* = 3614, 29%) and ‘bruises/swelling’ (*n* = 3341, 27%), with the least common being ‘amputation/crushing’ (*n* = 1). The proportions of injury by body site for each nature of injury is shown in Table 2.

**Table 2:** The proportions of injury by body site for each nature of injury

Nature of Injury	Sprains/Strains	Multiple Natures	Bruises/Swelling	Fractures/Dislocations	Occupational Overuse Syndrome	Other	Amputation/Crushing	Total
Body Site								
Leg Upper	62.2%	8.0%	29.2%	0.5%	< 0.1%	0.01%	< 0.1%	100%
Leg Not Classified	35.7%	17.8%	41.1%	5.4%	< 0.1%	< 0.1%	< 0.1%	100%
Hip	59.0%	5.5%	30.8%	1.2%	3.5%	< 0.1%	< 0.1%	100%
Groin	62.6%	4.1%	33.3%	< 0.1%	< 0.1%	< 0.1%	< 0.1%	100%
Knee	50.3%	15.7%	32.7%	1.2%	0.1%	< 0.1%	< 0.1%	100%
Leg Lower	45.3%	18.5%	32.3%	3.8%	0.1%	< 0.1%	< 0.1%	100%
Ankle	62.7%	8.9%	25.7%	2.6%	0.1%	< 0.1%	< 0.1%	100%
Foot	44.8%	6.8%	42.8%	4.8%	0.8%	< 0.1%	< 0.1%	100%
Toe/s	12.0%	12.0%	59.3%	14.8%	0.9%	0.10%	0.9%	100%
Multiple Locations	< 0.1%	5.7%	91.4%	2.9%	< 0.1%	< 0.1%	< 0.1%	100%
Other/ Unspecified	< 0.1%	< 0.1%	75.4%	21.1%	3.5%	< 0.1%	< 0.1%	100%
Multiple body sites (Only MSK)	36.6%	42.4%	20.2%	0.7%	0.1%	< 0.1%	< 0.1%	100%
Multiple body sites (including MSK)	23.6%	60.5%	15.3%	0.4%	0.2%	< 0.1%	< 0.1%	100%

There was a statistically significant, albeit weak [39] association (Cramer’s V = 0.106) between gender and the nature of injury (χ2[6] = 138.893, *p* < .001). The most common nature of injury, when comparing between genders, was found to be ‘sprains/strains’ for both male officers (*n* = 4060, 45%) and female officers (*n* = 1199, 35%). This was followed by ‘multiple natures including MSK’ (*n* = 2626, 29%) and ‘bruises/swelling’ (*n* = 2196, 24%) for male officers and ‘bruises/swelling’ (*n* = 1145, 34%) and ‘multiple natures including MSK’ (*n* = 988, 29%) for female officers.

When considering levels of reported injury severity, based on treatment received and lost work time, a substantial proportion of injuries were reported as ‘notification only with no time loss or treatment’ (*n* = 8573, 69%; meaning the injury was reported only to document the injury and associated risk or hazard and that no medical treatment was required and no time was lost from work), and this was followed by ‘medical treatment only - with no time loss’ (*n* = 1921, 15%), and ‘medical treatment only - with time loss’ (*n* = 1484, 12%). Only a small number of injuries required ‘hospitalisation’ (*n* = 429, 3%) and the remaining injuries (*n* = 45, 0.4%) were recorded as ‘treatment not stated’. The levels of injury severity recorded for male and female officers did not differ significantly, as shown by a weak [39] association (Cramer’s V = 0.028) between gender and injury severity categorisation (χ2[4] = 10.033, *p* = .040). For both male (*n* = 6257, 69%) and female officers (*n* = 2316, 68%) ‘notification only with no time loss or treatment’ was the most common level of severity, followed by ‘medical treatment only - with no time loss’ (male officers: *n* = 1408, 16%; female officers: *n* = 513, 15%), and ‘medical treatment only - with time loss’ (male officers: *n* = 1039, 12%; female officers: *n* = 445, 13%).

The most common locations at which injuries occurred were reported to be ‘at the workplace’ (i.e., at the station/lab/office or in a police vehicle; *n* = 4656, 37%), ‘at work but not on police premises’ (*n* = 4544, 37%), or ‘on patrol’ (*n* = 1915, 15%). The smallest numbers of injuries were reported to occur ‘at a crime scene’ (*n* = 590, 5%). There was a statistically significant, albeit weak [39] association (Cramer’s V = 0.137) between gender and the locations at which injuries occurred (χ2[4] = 232.195, *p* < .001). For male officers being ‘at work but not on police premises’ (*n* = 3438, 38%), ‘at the workplace’, (*n* = 3178, 35%), and ‘on patrol’ (*n* = 1556, 17%) were the most common locations at which injuries occurred. Conversely, for female officers the most common locations at which injuries occurred were ‘at the workplace’ (*n* = 1478, 44%), ‘at work but not on police premises’ (*n* = 1106, 33%), and ‘on patrol’ (*n* = 359, 11%).

‘Arresting offenders’ (*n* = 3012, 24%) was found to be the most common activity being undertaken at the time an injury occurred, followed by ‘walking/running’ (*n* = 1446, 12%) and ‘other/unspecified’ activities (*n* = 1139, 9%). ‘Motorcycle pursuits’ and ‘parking control’ were found to be the least common activities, associated with only three injuries in total (< 0.1%). When comparing between genders, there was a statistically significant, albeit weak [39] association (Cramer’s V = 0.236) between gender and the activity being undertaken at the time of injury (χ2[35] = 692.201, *p* < .001). The most common activity being performed at the time an injury occurred was ‘arresting an offender’ (*n* = 2450, 27%), followed by ‘walking/running’ (*n* = 838, 9%), and ‘foot pursuit’ (*n* = 837, 9%) for male officers and ‘walking/running’ (*n* = 608, 18%), followed by ‘arresting an offender’ (*n* = 562, 17%), and ‘general duties’ (*n* = 382, 11%) for female officers.

‘Slips/trips/falls’ (*n* = 4701, 38%), and ‘physical assault’ (*n* = 1839, 15%) were found to be the top two causes of injuries, followed by ‘other/unspecified’ causes (*n* = 1427, 12%). In relation to gender differences in percentages of injuries associated with slips, trips and falls, particularly, there was a statistically significant, albeit weak [39] association (Cramer’s V = 0.137) between gender and the cause of the incident (χ2[32] = 253.134, *p* < .001). The most common cause of injury for both male officers (*n* = 3204, 35%) and female officers (*n* = 1497, 44%) was ‘slips/trips/falls’ with little difference in the two following most common causes of injury, being ‘physical assault’ (male officers: *n* = 1369, 15%; female officers: *n* = 470, 14%), and ‘other/unspecified’ (male officers: *n* = 970, 11%; female officers: *n* = 457, 12%). These results suggest that female officers were more likely than male officers to incur an injury due to slips, trips and falls.

The most common duty types being undertaken by officers at the time of injury were found to be ‘general duties’ (*n* = 5924, 48%), ‘highway patrol’ (*n* = 1318, 11%), and ‘criminal investigator’ (*n* = 926, 7%). When comparing between genders, the most common duty type for both genders was ‘general duties’ (male officers: *n* = 4339, 48%; female officers: *n* = 1585, 47%), followed by ‘highway patrol’ (male officers: *n* = 909, 10%; female officers: *n* = 409, 12%), and ‘criminal investigator’ (male officers: *n* = 693, 8%; female officers: *n* = 233, 7%). However, while the most common types of duties being undertaken at the time of injury were very similar for male and female officers, their was a weak [39] association (Cramer’s V = 0.090) between gender and the duty type at time of injury (χ2[37] = 101.385, *p* < .001). These differences arose from variations in other duty types between the genders. For example, for female officers, 2.4% (*n* = 81) of injuries arose from water policing duties, while a lower 1.6% (*n* = 143) occurred for male officers completing these same duties.

## Discussion

The aim of this retrospective cohort study was to profile lower extremity MSK injuries within a state police agency. Lower extremity MSK injuries accounted for 19% of all reported work health and safety incidents. The overall MSK lower extremity incidence rates were 10.8 injuries per 100 personnel/year overall, 10.8 per 100 personnel/year for male LEO and 11.0 per 100 personnel/year for female LEO. The ‘knee’ was the body site of 31%, and ‘sprains/strains’ were the nature of injury for over 42%. For 69% of the reported lower extremity MSK injuries, the level of severity was reported as ‘notification only with no time loss or treatment’, and ‘the workplace’ was the most common location where the injuries were reported to occur (37%). ‘Arresting offenders’ was the most common activity being undertaken at the time injuries occurred (24%), ‘slips/trips/falls’ caused 38% of the reported injuries, and 48% of the injuries were reported to have occurred during the performance of ‘general duties’.

Similar to the findings of this study, other studies of LEO injuries by Sullivan and Shimizu [18] and Holloway-Beth et al. [40] found lower extremity injuries represented 13 and 30% of all reported workplace health and safety incidents, respectively. In the first study by Sullivan and Shimizu [18] 2167 descriptive injury claims, 417 retrospective worker’s compensation claims and 261 prospective claims were investigated from the Los Angeles county sheriff’s department. Similar to the current study, the retrospective data in that study showed the top site of lower limb injury to be the knee. The study of Holloway-Beth et al. was also completed in a law enforcement population, however the 18,892 claims in that study included claims from personnel of correctional institutions, municipal police, sheriff’s department personnel and state police. The differences in law enforcement populations, together with differences in definitions of incidents and injuries may account for the differences in proportions of incidents that were reported as lower extremity injuries across these three studies.

In other tactical populations, such as fire and rescue and the military, the lower extremity has been reported to be the body site affected in a larger proportion of incidents than in LEO. In fire and rescue populations the lower extremity is affected in 32% [7] to 37% [8] of reported injuries. In military populations, the lower extremity has also been reported to be the site of injury in a larger proportion of overall injuries [22] varying from 31.7% [6] to 82% [41] depending on the military population. Within U.S. Marine Corps training, injuries to the lower extremity can comprise up to 82% [41] of reported injuries. Other military populations such as U.S. Army male infantry trainees, Norwegian conscripts, U.S. Military non-deployed active duty personnel, Australian Defence Force personnel undertaking physical training and the overall Australian Defence Force population have reported the lower extremity to be affected in 80% [20], 63% [42], 39% [43], 48% [6], and 31.7% [6] of incidents, respectively.

In the aforementioned fire and rescue and military studies, it was not stated whether the lower extremity injuries reported were specifically MSK injuries and this may have contributed to the higher proportions of injuries involving the lower extremity when compared to the law enforcement population considered in the current study. Another contributing factor to this variation between studies in different tactical populations may be differences in occupational tasks, such as load carriage requirements, which can contribute to an increased injury risk [6, 9, 20–23]. LEO carry loads which can range from 3 kg to 15 kg [26] in general duties officers, normally consisting of duty belts and associated appointments, but they can also include body armour or additional equipment [23, 44, 45]. In specialist officers this can increase to loads of between 22 kg [27] and 40 kg [28], consisting of duty belts, body armour, specialist weapons, breaching equipment, ballistic shields and various personal protective equipment [23, 27, 28, 44–48]. Fire and rescue populations can carry loads ranging from 16 kg to 22 kg [49, 50]. Within the military, load carriage is often heavier than in the other two tactical populations, reaching 45 kg or more [49, 51].

In military populations, the most common lower extremity injuries have been reported to be the ankle, with percentages varying from 13% [43] to 37% [9], and the knee, which is affected in between 22% [9, 43] and 35% [22] of all lower extremity injuries. However, under-reporting of injuries within the military is common and the knee is the most frequently under-reported area, representing 20% of all unreported injuries [52]. Another recent study [53] also reported there to be a very high likelihood of substantial under-reporting (estimated at 80–90% of injuries unreported) on work health and safety incident reporting systems within military populations. Within the fire and rescue population the knee is the most common body site of injury, and it has been found to be affected in between 10% [54] and 14% [7] of all injuries, and in 45% [54] of lower extremity injuries. Accounting for the under-reporting within the military population, fire and rescue and military personnel appear to exhibit a relatively greater number of knee injuries than LEO. Reasons for this difference may include differing occupational tasks [31] - particularly load carriage requirements, which have been reported to cause knee injuries within military populations [55–58]. Greater load carriage demands in both military [49, 51], and fire and rescue [49, 50] personnel may increase the incidence of lower extremity injuries [6, 9, 20–23], particularly of the knee [55–58], in these populations.

The most common nature of injury was found in the current study to be sprains and strains (42.3%). Similarly a critical review of studies investigating law enforcement injuries by Lyons et al. [34], found that the most common nature of injury was also sprains/strains, varying from 42% [14] to 95% of reported injuries [16]. In the military population, sprains/strains have also been found to be the most common nature of injury, accounting for 80% of injuries [9]. Similarly, in the fire and rescue population sprains/strains were the most common types of injury, accounting for between 40 and 85% [8, 59–61] of all injuries. This is also the case in sporting populations such as athletes from weight-lifting sports [62], high school sports [63], ballet [64], and track and field [65].

The reported level of severity for the majority of lower limb MSK injuries (69%) in this study was “notification only with no time loss or treatment”. This was followed by “medical treatment only with no time lost” (15%). In total, 84% of all lower extremity MSK injuries resulted in “no time lost”. In the aforementioned critical review [34] of the literature focusing on the law enforcement population, sixteen articles [12–18, 40, 66–73] were included and in these studies treatment types and time loss, as indicators of severity, were not specifically reported. In the military study discussed [74], treatment was not reported whereas severity of injury was, with ‘minor injuries’ comprising approximately 57% of injuries, which is lower than the proportion of law enforcement injuries reported in the current study that involved ‘no time loss or treatment’ and so also could be considered ‘minor’. In the fire and rescue population, similar statistics to those for law enforcement were found - injuries were, where relevant, classified as minor, which was described as notification only, first aid only or medical treatment without time lost, representing 68% [54] to 96% of injuries [8]. The results of this study and from fire and rescue populations [54] suggest that the majority of incidents in these two populations were of a minor severity and involved no time lost, whereas in the military this proportion was substantially less although the minor injury contribution still represented over one half of injuries. These differences between populations may be attributed to deployed environments and combat related injuries causing higher severities of injuries within the military population as well as the aforementioned underreporting in military contexts, which may be more likely with minor injuries.

The most common locations at which injuries occurred were reported to be at the workplace (37%) and at work but not on police premises (37%). With no other LEO studies reporting the locations at which injuries occurred, comparisons were limited to military and fire-fighter populations. In contrast to the findings of this study, the military population attributed the majority of injuries to playing individual or team sport (64%), followed by completing physical training (20%) [9], both of which may, or may not, have occurred at the workplace. Similar to law enforcement in the fire and rescue population, 59% of injuries occurred at a fire station, whilst 36% occurred whilst at work but not within the station itself, and 4% were attributed to work related travel [7]. When comparing this particular element of injury profiling it is important to note that in the military, physical training may have been mandatory and as many members participate in physical training and sports as part of their military duties, this workplace requirement may account for the variation in the locations at which injuries occur when compared to other tactical populations.

Arresting offenders (24%) was found to be the most common activity being undertaken by LEO at the time they were injured, followed by walking/running (12%). In contrast to this study, a study by Larsen et al. [16] in a LEO population found that over half of the injuries reported in their study occurred during police specific occupational tasks, whilst greater than 30% occurred during training. Differences between the common activities associated with injuries in these studies, which both used data from state LEO in Australia, may be due to the current study reporting only on MSK lower extremity injuries for all officers, and the other study reporting only on a specialist unit and on all injuries. In the military population it has been reported that most injuries occur within military training [6, 9, 21, 22, 41–43, 53], although one study reported 33% of injuries occurred whilst walking/jogging [9]. Within the fire and rescue population two studies [60, 75] found that the most common activity at the time of injury was training/physical exercise (including walking/running), which in one study accounted for 33.3% of injuries [60]. In contrast, another study [59] reported the most common activity at time of injury to be fire and rescue specific occupational demands. However, over 30% of reported injuries in this study were still attributed to physical training [59]. Differences in activities associated with injuries relate directly to the types of occupational tasks. Arresting offenders is predominantly a law enforcement specific task and therefore is unlikely to be seen in other tactical populations to a great extent (e.g it might be seen in military police) if at all. Interestingly, physical exercise/training, which likely involves walking/running, appears in the top two activity types associated with injuries across all three tactical populations.

Slips/trips/falls (37.8%) and physical assault (14.8%) were found to be the top two causes of lower extremity MSK injuries for this state law enforcement population. Within military populations, the most common cause of lower limb injuries is over-exertion, both acute over-exertion and cumulative loading, which account for 59% of injuries, with slips/trips/falls causing 19% [9]. In fire and rescue populations, muscular stress both without external load and with an external load accounted for 29.7% of injuries, while slips/trips/falls accounted for 15.1% of injuries [7]. Slips/trips/falls contribute to a larger proportion of injuries within LEO when compared to military and fire and rescue populations. Reasons for this may be increased load carriage demands in the other two occupations, causing muscular stress/exertional injuries [21, 76].

The most common duty type in which injuries occurred in the LEO was general duties (47.6%), followed by highway patrol (10.6%). These duty types are specific to the law enforcement occupation and are therefore unable to be compared with other tactical populations. Likewise, the nature of law enforcement work produced some interesting results in relation to when injuries occurred during a shift. Significantly more injuries occurred during the middle third of the shift, although the actual differences in injuries reported between early and mid-shift was approximately 1% (*n* = 116 fewer injuries in the early third of the shift). A potential reason for this difference may be the administration associated with the ending of a shift (e.g. Hand over / Take over briefings) and hence the lower percentage of injuries reported (26%) in the last third of a shift but further research is needed to support this supposition.

Differences between the genders did exist in several areas notably in location (i.e., in the workplace) and activity being performed (i.e., walking/running). In addition, female officers may be at a higher risk of slips, trips and falls. As such targeted injury prevention strategies may have different impacts between genders. For example, strategies to minimise injuries caused by ‘physical assault’ could reduce the incidence of these injuries equally for both female and male officers (currently 14 and 15% respectively). However, strategies to mitigate the risk of injury due to ‘slips, trips and falls’ may reduce the incidence of injuries to female officers to a greater extent than male officers (currently 44 and 35% respectively). Of note, no differences in the severity of injuries between the genders were reported. This contrasts the findings of Orr et al. [35], who found that while male and female soldiers suffered similar levels of injuries due to occupational load carriage, female personnel were 2.4 times more likely to suffer a ‘serious personal injury’ than male personnel. Noting that the research by Orr et al. [35], was of a single task (load carriage) in a military population, potential differences in severity are reported by Hua et al. [77], who found that female police officers, in general, attended a greater number of in-house physiotherapy treatments than male officers (8.25 ± 5.12 vs. 6.57 ± 4.03 treatments respectively). Differences in these findings may be explained by several factors including, a lack of detail in regard to rating the severity of the injuries entered into the database, differences in reporting processes (e.g., self-reported, as was the case in this study) versus point-of-care (i.e. those attending physiotherapy treatment) [36].

The limitations to this study included; 1) that the classification systems employed in the original data were not very detailed, limiting information that could be derived from the dataset to guide injury prevention, 2) that the study focused on lower limb MSK injuries – other injury and incident types were outside the scope of the study.

The study also has several strengths. The data available were extensive, spanning 7 years and thousands of injury records. This study employed a standardised injury definition, which will enable future comparison across other tactical populations, where previously multiple different definitions have been used or no definition was provided. Finally, this study reports on a wide variety of key injury variables, including nature of injury, type of treatment, location at time of injury, activity at time of injury, cause of injury and the duty type, and the injured body site.

## Conclusion

Establishing the current profile of lower extremity injuries within a state law enforcement agency will address some of the current key limitations in the literature and enable ease of comparison to other occupations, government agencies and other tactical populations. This study found differences between LEO and other tactical populations in their profiles of lower extremity injuries, which may be due to the unique challenges LEOs face in their occupation, for example, arresting offenders, which was the most common activity being undertaken at time of injury. The most common site of lower extremity injury was found to be the ‘knee’, the most common nature ‘sprains/strains’, and the most common location of where the injury occurred was ‘at the workplace’ and ‘at work but not on police premises’. These findings can be used to inform the future development of injury prevention protocols, resilience testing and current physical testing protocols.

## Data Availability

The data that support the findings of this study are not openly available given security restrictions within the police agency from which this data is drawn. However, researchers can apply for access to this data, and on meeting all ethics requirements bound by this research, a reach forward request to the police agency to release the data can be made.

## Abbreviations

MSK: Musculoskeletal
CDC: Centre for Disease Control and Prevention
LEO: Law enforcement officers
BUHREC: Bond University Human Research Ethics Committee
SD: Standard deviations
